# Postradiation Histiocytic Sarcoma in the Setting of Muir-Torre Syndrome

**DOI:** 10.1155/2018/5947870

**Published:** 2018-04-24

**Authors:** Erin Baumgartner, David Ullman, Jeffrey Adam Jones, Danielle Fasciano, Daniel S. Atherton, Peter Pavlidakey, Deniz Peker

**Affiliations:** ^1^Department of Pathology, UAB, Birmingham, AL, USA; ^2^Department of Dermatology, UAB, Birmingham, AL, USA

## Abstract

Hereditary nonpolyposis colorectal carcinoma (HNPCC) is an autosomal dominant genetic disorder characterized by a predisposition towards colorectal carcinoma and other extracolonic neoplasms. Histiocytic sarcoma (HS) is a very rare hematologic neoplasm characterized by a malignant proliferation of cells with histiocytic differentiation. We present the case of a 62-year-old male with previous diagnosis of MTS who presented with metastatic colorectal adenocarcinoma, bilateral papillary renal cell carcinoma, and a new squamous cell carcinoma of the scalp, treated with resection and adjuvant radiation therapy. After reconstructive surgery for his scalp resection, the patient developed a persistent nonhealing skin defect. A punch biopsy of this nonhealing skin defect and subsequent immunohistochemistry revealed neoplastic histiocytic cells restricted to the epidermis and underlying dermis. The diagnosis of cutaneous histiocytic sarcoma was then rendered. Histiocytic sarcoma is an exceptionally rare malignancy. Consequently, there is no universally agreed upon management protocol for this malignancy. The patient was admitted to hospice and treated with palliative radiation. This case demonstrates the need for awareness of the risk of secondary malignancies in cancer patients in order to facilitate early surgical intervention and optimal treatment.

## 1. Introduction

Hereditary nonpolyposis colorectal carcinoma (HNPCC), also known as Lynch syndrome, is an autosomal dominant genetic disorder with a high degree of penetrance and variable expressivity caused by a constitutional defect in a mismatch repair (MMR) gene, most commonly affecting the genes MLH1, MSH2, MSH6, and PMS2 [1–3]. This syndrome is characterized by a predisposition towards the development of colorectal carcinoma and various other extracolonic carcinomas that may involve the endometrium, ovaries, stomach, small bowel, and pancreas [2, 4–6]. When these extracolonic neoplasms also include the formation of sebaceous neoplasms, such as sebaceous adenoma or sebaceous carcinoma, the HNPCC diagnosis is further categorized as Muir-Torre Syndrome (MTS) [5]. Interestingly, there have been reports in the literature of HNPCC occurring in association with hematologic malignancies and sarcomas [7, 8]. An exceptionally rare hematologic neoplasm, known as histiocytic sarcoma (HS), is characterized by the proliferation of malignant cells of histiocytic differentiation [9]. To our knowledge, the current case represents the first occurrence of histiocytic sarcoma appearing in conjunction with underlying MTS, as well as the second case of a postradiation sarcoma appearing in the setting of this rare syndrome [10].

## 2. Case Report

**Figure 1:**
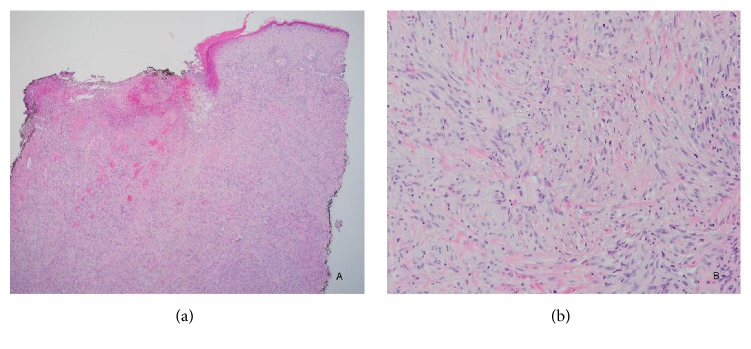
(a) Punch biopsy reveals a spindle cell lesion infiltrating the dermis (H&E, 40x); (b) (H&E, 200x).

**Figure 2:**
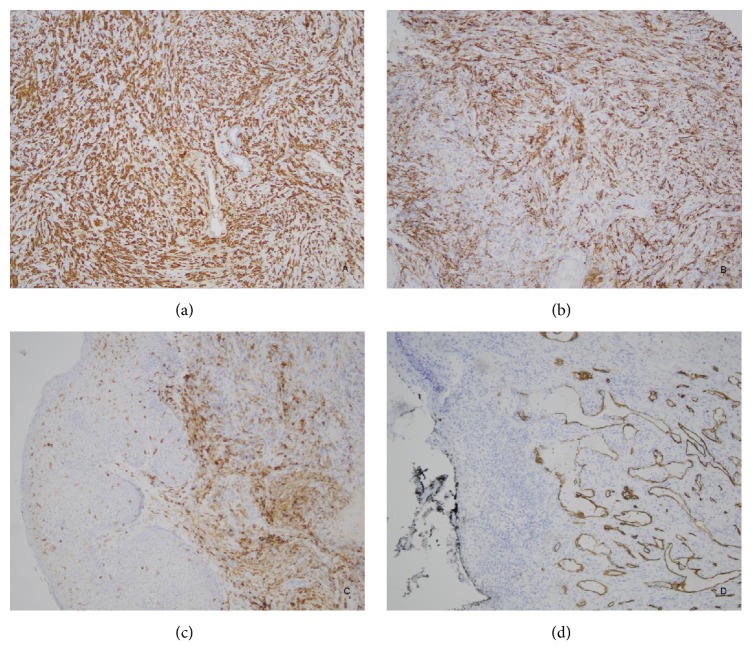
Immunohistochemistry was positive for (a) CD 163+, (b) CD14+, and (c) CD4+. CD163 and CD14 are expressed on macrophages, while CD4 is expressed on other cells, such as helper T-cells; CD4 is also expressed on monocytes/macrophages. Immunohistochemistry was negative for (d) CD34. CD34 negativity indicates that this neoplasm is not a myeloid sarcoma.

The case reported is of a 62-year-old male with a complicated past medical history. He received a bilateral kidney transplant, the first in 1990 and the second in 2006, for focal segmental glomerulosclerosis followed by bilateral papillary renal cell carcinoma in 2008, for which he had bilateral native nephrectomies. He presented to UAB with invasive colorectal adenocarcinoma in 2010, and in 2011 he was diagnosed at UAB with Lynch syndrome/MTS with a documented mutation of the MSH6 gene. A year later, he presented with a recurrence of colorectal adenocarcinoma, now metastatic to the liver and retroperitoneum. Additionally, the patient had an extensive history of recurrent squamous cell cancer, with multiple lesions removed almost monthly. In 2016, he presented with a presumed new squamous cell carcinoma of the scalp, which was treated with resection and adjuvant radiation therapy. He subsequently developed a nonhealing skin defect. Initial clinical impression of this skin defect favored a diagnosis of a recurrent squamous cell carcinoma. A punch biopsy was performed and microscopic examination revealed unremarkable surgical site changes with adjacent actinic keratosis. The scalp defect was treated with excision and reconstructive surgery (bilateral fasciocutaneous flaps and full thickness skin grafts). After reconstructive surgery, the patient developed a nonhealing nodule at the surgical site, measuring 1 cm in diameter. A punch biopsy of this new lesion revealed sheets of polygonal and pleomorphic atypical cells with a focal storiform architecture, as well as abundant necrosis and increased mitotic activity (Figure 1). On immunohistochemical (IHC) stain examination, the neoplastic cells were reactive only for CD163, CD14, and CD4 (Figure 2). The possibility of a myeloid sarcoma was ruled out based on the malignant cells' failure to stain with CD43 and CD34. The lesional cells were also negative for CDX2, anti-HMW cytokeratin (34bE12), CAM5.2, desmin, SMA, caldesmon, CD21, SOX10, pancytokeratin, ERG, p63, and S100, effectively ruling out poorly differentiated carcinoma, sarcoma, and melanoma. A diagnosis of histiocytic sarcoma was rendered based upon positive staining for histiocytic markers such as CD163 and CD68, as well as the T-lymphocyte marker CD4, which can also be expressed in cells of the myelomonocytic and histiocytic lineage [11]. The patient was admitted to hospice and treated with palliative radiation.

## 3. Discussion

Although neither hematopoietic neoplasms nor sarcomas are traditionally considered part of the HNPCC/MTS criteria, Sijmons et al. proposed the inclusion of malignant fibrous histiocytoma (MFH) in the tumor spectrum of HNPCC [7]. DNA analysis of one MFH arising in a background of MTS revealed microsatellite instability and the loss of one MSH2 copy [7]. Immunohistochemistry staining showed an absence of nuclear MSH2 protein, further indicating a relationship between the presence of HNPCC in the patient and the new tumor [7]. After noting additional case reports of several HNPCC families with an increased incidence of lymphomas and acute leukemias, Bandipalliam proposed the formation of a subtype of HNPCC, categorized by features of neurofibromatosis and the presence of concurrent hematologic malignancies [8]. Although there may be an increased incidence in hematologic malignancies in HNPCC patients, this, to our knowledge, is the first reported case of histiocytic sarcoma arising in the setting of HNPCC/MTS [10].

**Figure 3:**
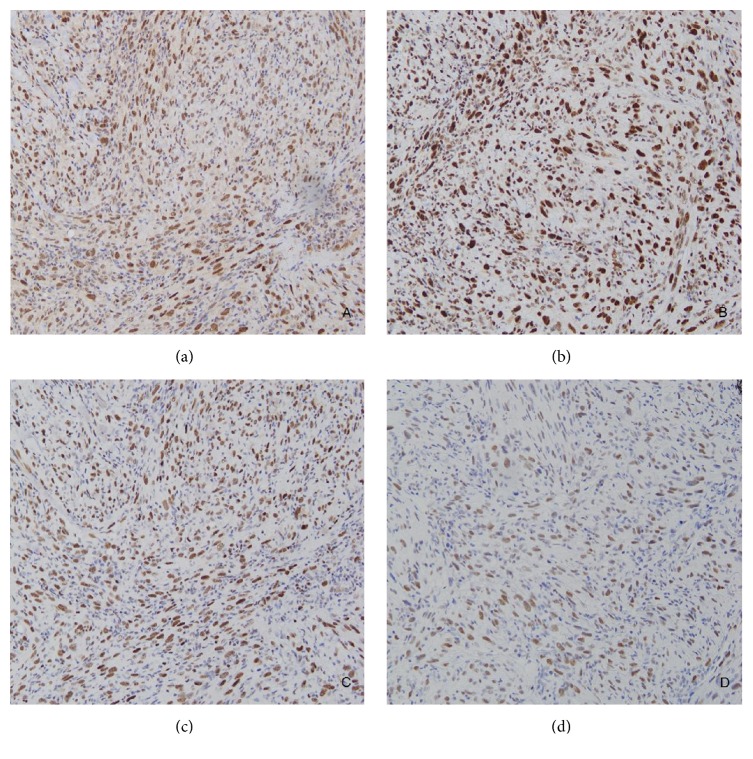
Immunohistochemistry showed positive staining for (a) MLH1+, (b) MSH 2+, (c) MSH6+, and (d) PMS2+. Positive staining indicates the absence of microsatellite instability in this neoplasm.

In the vein of Sijmons et al., we stained our patient's tumor with microsatellite instability markers [7]. Despite the patient's history of a documented mutation of the MSH6 gene, the sample was positive for MLH1, MSH2, MSH6, and PMS2, indicating the absence of microsatellite instability (Figure 3). Although IHC and MSI are considered to be sensitive prescreening tools for the detection of MMR gene defects, with a correspondence of almost 100% between both tests, the presence of MSI is not reflective of the specific gene defect present, and the maintenance of expression of the MMR proteins on IHC does not exclude the possibility of an underlying DNA repair defect [3]. A study conducted by Cadéés et al. showed that the mutations in MSH6 do not always produce microsatellite instability (MSI) in the tumors of HNPCC. Additionally, relatively young lesions are likely to show less microsatellite instability, with rare cases of HNPCC even presenting as microsatellite stable (MSS) [1]. However, this immunohistochemical profile would seem to suggest that there was no association between the patient's underlying MTS and the histiocytic sarcoma.

Alternatively, rather than the presence of an underlying HNPCC/MTS, the pathogenesis of the patient's HS may have been initiated by radiation. It has been hypothesized that certain populations of cells are susceptible to the development of therapy-related myelodysplastic syndrome (TR-MDS) due to impaired DNA damage repair secondary to therapeutic radiation or the inability to metabolize chemotherapeutic agents [12]. To our knowledge, there is only one other documented case of a postradiation HS in the literature [13].

With only a few hundred cases of histiocytic sarcoma reported in the literature, this is the first documented case of this entity arising in an MTS patient. Although theoretically one might suspect the patient's MTS to have predisposed him to the development of HS, immunohistochemical staining indicates that the two events were likely unrelated. This finding and the fact that HS has never before been documented in a case of an MTS patient lend some credence to the notion that histiocytic sarcoma should not be included in the MTS associated tumor spectrum. Regardless, this is a unique case that demonstrates the need for increased awareness of the risk of secondary malignancies in cancer patients.
